# Onco-immunity and therapeutic application of amygdalin: A review

**DOI:** 10.1016/j.jobcr.2022.12.010

**Published:** 2022-12-28

**Authors:** Ahmed Mohammed Alwan, Dinesh Rokaya, Goma Kathayat, Jalil Tavakol Afshari

**Affiliations:** a Department of Immunology and Allergy, Faculty of Medicine, Mashhad University of Medical Sciences, Mashhad, Iran; b Department of Clinical Dentistry, Walailak University International College of Dentistry, Walailak University, Bangkok 10400, Thailand; c Department of Biochemistry, Manipal College of Medical Sciences and Teaching Hospital, Pokhara, Nepal

**Keywords:** Amygdalin, Beta-glucosidase, Cancer, Therapeutic application, Herbal remedy

## Abstract

**Background:**

Amygdalin is known as a chemical compound derived from various fruits. The glycosides existing in this plant have been historically utilized as an anticancer agent. This review presented an overview of amygdalin and its onco-immunity and other therapeutic medical applications.

**Method:**

A literature search for studies relating to amygdalin and cancer treatment was carried out using PubMed and Google Scholar. Combinations of the following terms were used in the search strategies: “amygdalin,” “rhodanese,” “cyanide,” “cyanogenic,” “hypothiocyanite,” “mandelonitrile,” “glucosides,” “cancer,” “apoptosis,” and “cytotoxicity,” combined with a cancer term such as “seed,” “almond,” or “apricot,” “cancer + cell line, antiproliferation or inhibition,” “BAX From the March 3, 1981 until the April 15, 2021, all of the English-language papers were evaluated based on the inclusion criteria. Publications included reviews, chapters from books, and original research papers.

**Results:**

The FDA prohibits Amygdalin from medical usage as an anticancer treatment due to a lack of proof of cure in cancer cases. When this natural-based compound is used with conditional chemotherapeutic medicines causes synergistic effects. Besides, amygdalin is used to manage asthma, improve the immune system, induce apoptosis in human renal fibroblasts, and inhibit hyperglycemia.

**Conclusion:**

Various medical uses of amygdalin have been found such as managing asthma, improving the immune system, inducing apoptosis in human renal fibroblasts, and inhibiting hyperglycemia. More effective in vitro and review studies are required to elucidate the exact role of this herb in medical applications.

## Graphical abstract

**Figure:**
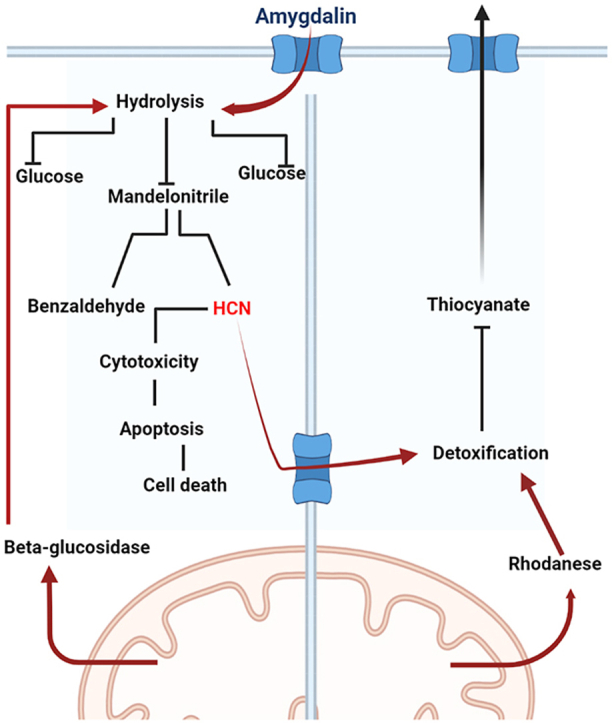

## 1 Introduction

Cancer is a heterogeneous disorder that comes from aberrant cell growth, differentiation, and death. It is also regarded as a serious issue in modern medicine.^1^ In normal cells, cell division, normal cell death, and differentiation are constantly in balance, but cancer cells are isolated from normal cell division, growth, and unrestricted proliferation.^2^ The specific source of this occurrence is unknown; however, hereditary factors or elements that alter cell function and the nucleus might be blamed.^3^ Radioactive substances, toxins, and chemical compounds, as well as excessive radiation such as sunshine, are among these.^4^ The second-largest cause of death is cancer worldwide.^5^ The American Cancer Society publishes statistics on new cancer deaths, cancer patients, and cancer prevalence and survival rates every year. Since 1991, the prevalence of cancer has reduced by around 23%, according to statistics.^6^ The death rate for malignancies of the liver, pancreatic, and uterine, on the other hand, is gradually growing. Several pharmacological investigations have shown that many herbal remedies have potent anticancer properties.^7^ Patients who have cancer frequently attempt to find “alternative” or “complementary” (CAM) therapy methods to actively assist their treatment process, minimize the chance of cancer recurrence, or lessen the negative effects of conventional therapies.^8^ Some complementary and alternative medicine include consuming natural products, homeopathy, mind-body therapies, traditional Chinese medicine, or Ayurvedic medicine. One of the most common CAM approaches is the intake of natural plants, employed in approximately half of the cases.^9^ Despite the extensive use of natural plants, there is little knowledge regarding how they can be used.

It should be noted that the natural substance amygdalin (D-mandelonitrile-b-D-glucoside-6-b-glucoside) is extracted from Rosaceae nuclei. It is a cyanogenic glycoside that can be found in various fruits.^10^ Amygdalin, found mostly in bitter almonds, has been shown to have anti-tumor capabilities in addition to antioxidant, antibacterial, anti-inflammatory, and immune-regulating characteristics.^11^ It has been demonstrated to have anticancer effects on solid tumors, namely bladder cancer, renal cell carcinoma, and lung cancer, via producing cytotoxicity and apoptosis, affecting the cell cycle, and influencing immunological function.^12^ The amygdala is regarded as a natural cancer therapy by its proponents.^13^ Even though the Federal Bureau of Drugs and Medicine (BfArM) has over 40 web connections to amygdalin therapy, the exact number of people using amygdalin is not clear.^14^ Amygdalin's opponents pinpoint its inefficiency and highlight that beta-glucosidase converts amygdalin to cyanide, resulting in severe cyanide poisoning.^15^ Beta-glucosidase levels in malignant tissues are more significant than normal levels in the liver and intestines. Since cancerous tissues have relatively low levels of the enzyme rhodanese compared to the liver and kidney, they can be targeted by cyanide secretion via the action of beta-glucosidase on amygdalin.^16^ In addition, amygdalin mostly shows vitamin-like effects. Mice nurtured with cyanogenic glycoside-free diets for numerous generations had no signs of tumors.^17^ Experimenting with amygdalin on tumor-resistant animals has demonstrated that it has therapeutic potential.^18^ On the other hand, amygdalin is nontoxic when taken orally; however, it is poisonous when combined with plant-rich beta-glucosidase.^19^

## 2 Method

A literature search for studies relating to amygdalin and cancer treatment was carried out using PubMed and Google Scholar. Combinations of the following terms were used in the search strategies: “amygdalin,” “rhodanese,” “cyanide,” “cyanogenic,” “hypothiocyanite,” “mandelonitrile,” “glucosides,” “cancer,” “apoptosis,” and “cytotoxicity,” combined with a cancer term such as “seed,” “almond,” or “apricot,” “cancer + cell line, antiproliferation or inhibition,” “BAX From the March 3, 1981 until the April 15, 2021, all of the English-language papers were evaluated based on the inclusion criteria. Publications included reviews, chapters from books, and original research papers. There were also in vitro studies, in vivo animal studies, or human studies, as well as clinical studies and meta-analyses. This review presented an overview of amygdalin and its onco-immunity and other therapeutic applications in biomedicine.

## 3 Origin of amygdalin

Robiquet and Bourton-Chalard initially extracted amygdalin from bitter almonds in the 1830s, and it was subsequently shown to be a cyanogenic glycoside in fruits.^20^ Amygdalin is also found in olive, grape, and buckwheat seeds, and it is present in the kernels of some fruits such as apricots (8%), peaches (6%), bitter almonds (5%), and plums (2.5%). Besides, apple seeds, lima beans, clover, and sorghum all contain amygdalin.^21^ Amygdalin is thought to be a plant-derived chemical, with the laetrile form as the refined version.^22^ In the 1950s, Krebs produced amygdalin as an injectable drug and patented it as lateral (laevorotatory mandelonitrile) for cancer therapy, causing confusion between amygdalin and amygdalin laetrile, laetrile, and d-mandelonitrile-glucuronoside glucose.^23^ There is still some uncertainty concerning these names. Mandelonitrile, which is made up of the cyanide group, is a primary component of both drugs.^24^ In the 1950s, amygdalin was referred to as “vitamin B-17,” while beta-d-glucosidase catalyzes the degradation of amygdalin.^25^

## 4 Amygdalin's structural properties and its biosynthesis

**Fig. 1:**
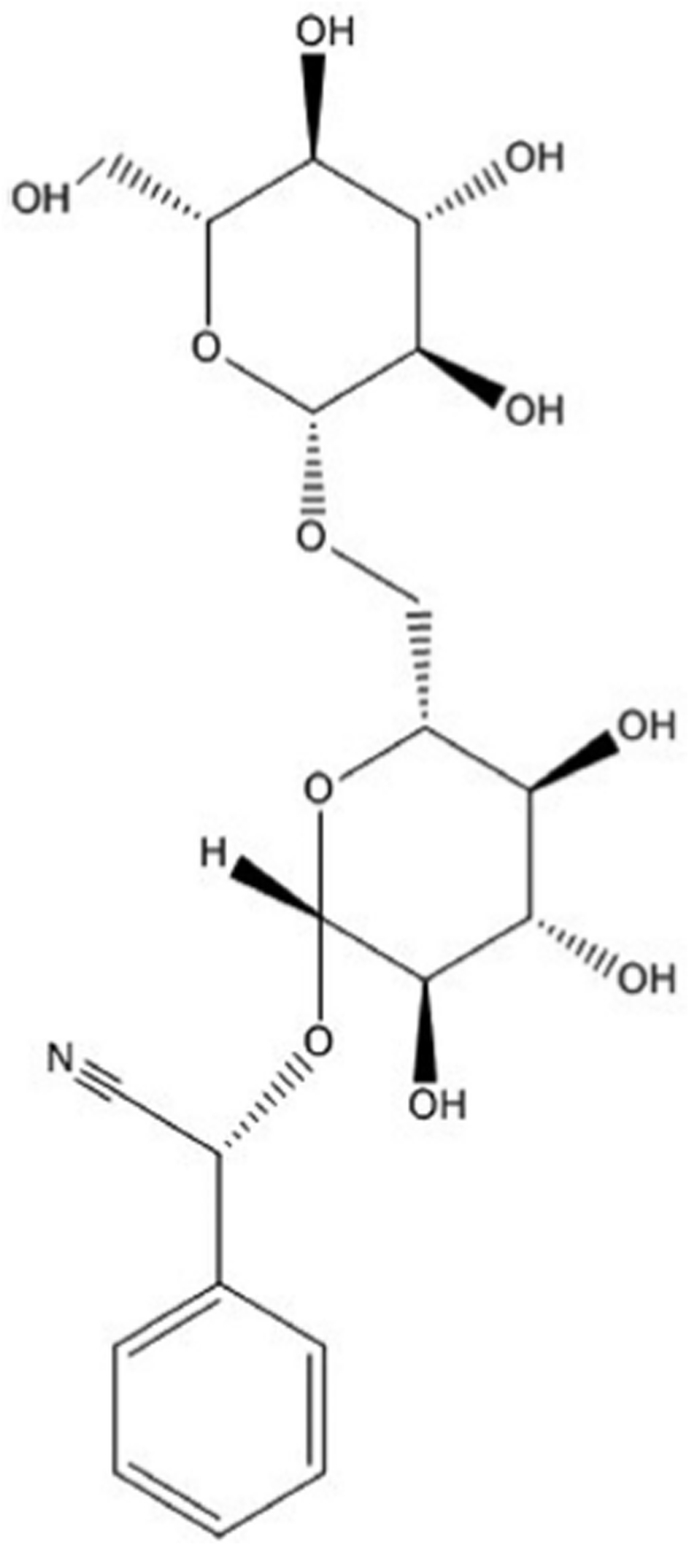
Amygdalin's chemical structure.

Amygdalin is a benzaldehyde, hydrocyanic acid, and glucose-containing aromatic aminoglycoside with the chemical formula of C20H27NO11 (Fig. 1).^26^ The chemical name for amygdalin (R) -α - [(6-O-β-D-glucopyranosyl-b-D-glucopyranosyl-oxy)] - (phenyl) is acetonitrile, also known as [d - (−) mandelonitrile-β-d-gentiobioside].^27^ The amygdalin's right-handed structure of R, its natural form, is also the active form of amygdalin.^28^ It is a colorless substance with a molecular mass of 457.4 g/mol, a melting point of 213 °C, and a chemical identification number (CAS) of 29883-15-6. It is insoluble in chloroform, a non-polar solvent; however, it is moderately soluble in water and highly soluble in ethanol.^29^ Amygdalin, laetrile, or vitamin B-17 are all referred to as the same substance. However, they are not interchangeable terms. Laetrile is the pure version of amygdalin, which is a cyanogenic glucoside.^30^ On the other hand, laetrile is a semi-synthetic cyanogenic glucuronide that differs from amygdalin in terms of structure. In the United States of America and Mexico, the synthesis of laetrile completely varies.^31^ Laetrile is a human-produced amygdalin in the US, while it is manufactured in Mexico from chopped apricot kernels.^32^ Laetrile is a vitamin or dietary supplement made by E.T. Krebs Jr., who developed the phrase vitamin B-17.^5^

The amino acid phenylalanine is hydroxylated to phenyl acetaldoxime in amygdalin by the enzyme CYP79, which is then hydroxylated to mandelonitrile (2-hydroxy-2-phenyl acetonitrile) by the enzyme CYP71.^8^ Mandelonitrile is the aglycone moiety of the cyanogenic glycosides prunasin and amygdalin.^9^ The formation of prunasin is caused by the subsequent binding of a glucose molecule to the -hydroxyl mandelonitrile group, which is catalyzed by the uridine glucose-glucosyltransferase uridine.^10^ Eventually, it is transformed into amygdalin by adding another glucose molecule to the 6′-hydroxyl group, generating the gentiobiose diglucoside.^12^ Amygdalin's enzyme hydrolysis generates benzaldehyde and hydrocyanic acid, whereas its acid hydrolysis generates gentiobiose as a single product.^13^ Amygdalin has pre-hepatic metabolism properties that result in prunasin production in the gut.^14^ It is questionable whether amygdalin increases tumor recurrence or is toxic in the correct quantity, and whether converting it leads to a poisonous chemical rather than a therapeutic medicine or not.^33^ Many laboratory tests have revealed that this chemical can kill malignant cells and block or slow the cell cycle course in various cancer cells.^34^ In addition, limited in vivo studies on the anticancer effects of amygdalin have indicated the inhibition role of the HeLa cell proliferation in nude mice.^35^ Studies on the toxicity of amygdalin revealed its toxicity, particularly when given orally, leading to its FDA prohibition in 1979.^36^

Furthermore, the intestinal anaerobic bacteria family can release cyanide in the gut as a result of the amygdalin breakdown.^37^ Rhodanese, a mitochondrial enzyme present in many species, may convert high amygdalin-derived cyanide to non-toxic thiocyanate.^30^ Amygdalin also has a variety of pharmacological actions, including analgesic and asthmatic properties.^38^

## 5 Metabolism of amygdalin

In 1967, Heismann and Knight published their first report on the full enzymatic and acid hydrolysis of amygdalin.^38^ Glucose and prunasin are formed when amygdalin interacts with beta-glucosidase. Prunasin is further hydrolyzed to obtain glucose, and another compound called mandelonitrile.^37^^,^^39^ This product is transformed into hydrocyanic and benzaldehyde acid without any enzymes.^40^ The acid hydrolysis of amygdalin yields a single genetic product (a disaccharide with 6-6 beta-binding compounds).^41^ The Michaelis–Menten kinetics can be utilized to identify the enzymatic processes involved.^42^ Three enzymes, amylase lyase, prunasin lyase, and hydroxyl lyase, have been found and catalyzed in three separate phases.^32^^,^^43^ Hydrocyanic acid and glucosidase are the two primary contributors to amygdalin for causing apoptosis and limiting cancer cell growth.^44^

Moreover, in the presence of lactate produced by cancer cells during anaerobic respiration, glucosidase performance is considerably enhanced.^45^ HCN can also kill cancer cells by raising their acidity and forming lysosomes to release their enzyme content, resulting in cell lysis.^46^ Amygdalin was detected in the circulation of mice 5 min after injection.^47^ Amygdalin has two metabolic routes, according to pharmacokinetic research.^41^ The first is the conversion of amygdalin to prunasin, which occurs before the liver or the first transit in the proximal intestine.^30^^,^^48^^,^^50^ Amygdalin metabolism in simulated gastrointestinal cell culture revealed that it is first degraded to prunasin, then to mandelonitrile.^49^ Subsequently, it is hydroxylated to hydroxy semandononitrile in the small intestine by beta-glucosidase.^47^ At this point, neither cyanide nor benzaldehyde is created, indicating that cyanide is most likely to develop in the lower intestine, densely populated with bacteria.^31^ In order to assess amygdalin content in blood, liquid chromatography-mass spectrometry was used (LC-MS) a specific method for detecting.^51^

**Fig. 2:**
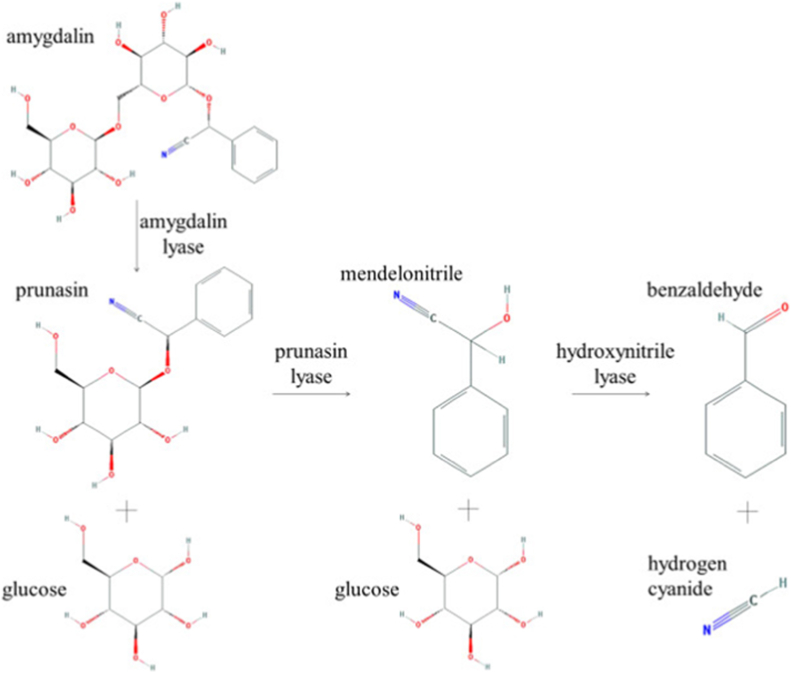
Metabolism of amygdalin.

According to various studies, amygdalin undergoes enzymatic hydrolysis and is transferred into two molecules of glucose and mandelonitrile, which is automatically changed to HCN and benzaldehyde due to its fragile nature as shown in Fig. 2.^37^ It may be oxidized further to benzoic acid, and if amygdalin is consumed orally, HCN can induce mitochondrial toxicity by blocking the enzyme cytochrome oxidase (ETC). In the presence of heat, the enzyme hydrolysis of amygdalin has been found to speed up. In manufacturing.^52^ For instance, fruits like cherries hydrolyze benzaldehyde by raising the rate of amygdalin hydrolase, prunasin lyase, beta-glucosidase, water, and mandelonitrile lyase when the temperature is raised to 65 °C.^53^

## 6 Pharmaceutical applications of amygdalin

Amygdalin's breakdown, which creates hydrocyanic acid, can slow down respiratory motions and treat asthma by blocking the respiratory system.^54^ An experimental animal model with respiratory illness conditions showed an increase in the synthesis of pulmonary surfactant.^55^ Amygdalin has been shown to protect type II alveolar epithelial cells (AECIIs) separated from young mice's lungs when subjected to hypoxia.^56^ This situation stops AECII cells from proliferating and lowers lung surfactant mRNA levels in vitro, resulting in lung injury in preterm animals.^57^ At a concentration of 200 μmol/L, amygdalin stimulates the proliferation of AECII cells in preterm mice and increases the levels of SP mRNA.^58^

Besides, Semen Armeniacae Amarum (SAA), which contains amygdalin as an active constituent, has an anti-asthma effect in an allergic asthma model by inducing ovalbumin (OVA).^59^ SAA is thought to decrease type 2 helper T cell function and diminish interleukin (IL-4) production.^60^ The consequences of asthma, such as airway hyperactivity (AHR) and airway inflammation, are reduced due to altered Th2 responses to the allergen. Reduced airway hyperactivity and inflammation, which are both asthma symptoms, might result from altered Th2 responses to the allergen.^61^ In addition, amygdalin suppresses acute lung damage produced by lipopolysaccharide. Amygdalin may also be used to treat chronic obstructive pulmonary disease (COPD).^62^ Several studies show that amygdalin can partially suppress epithelial-mesenchymal transmission generated by smoking.^63^ This impact might be attributable to amygdalin's capacity to reduce TGF-B1 expression and smad2/3 phosphorylation, linked to TGF/Smad pathway suppression.^64^

## 7 Amygdalin and the immune system

Amygdalin indicates an anti-atherosclerotic impact by decreasing the anti-inflammatory response and boosting the immune system by modifying regulatory T cells' action (Tregs), resulting in plaque elimination and an expanded lumen.^65^^,^^66^ Psoriasis can be effectively treated with T-peptide (PT), an octapeptide that is sometimes referred to as the amygdalin analog due to comparable peptide chains.^67^ In human keratinocytes, PT has been demonstrated to increase TGF-β, HSP70, and α-v integrin expression while decreasing ICAM-1 expression.^68^ Besides, three amygdalin-derived analogs with comparable medicines in the same peptide chain but no cyanide group were shown to have biological activities identical to PT.^69^ They both modulate the immune system in human keratinocytes, suggesting that they might be used to treat psoriasis.^70^

Amygdalin increases the growth of human peripheral blood T cells in vitro (25–800 μg/mL, 0.055–1.750 mmol/L).^35^ It can also boost IL-2 and interferon-secretion in the range of 25-400μ g/mL (0.055–0.875 mmol/L).^71^ Amygdalin (100–400 mg/L; 0.219–0.875 mmol/L) induced T cells to proliferate in additional trials, with the most impact at 200 mg/L (0.437 mmol/L). Amygdalin (10 mg/kg) reduces the growth of immune cells, suppresses the immune system, and increases the survival of kidney transplant mice in vivo.^72^ Immune cells have a vital role in the growth and development of endometriosis. Amygdalin (5 mg/kg) lowers endometriosis foci by affecting immune cells' local activity.^48^ These findings indicate that amygdalin can promote the proliferation of immune cells in lab studies, and simultaneously, it can inhibit the proliferation of immune cells and enhance organ transplant success rates in organ transplant tests.^55^ These two seemingly contradicting findings point to a possible amygdalin-mediated immune system regulation on both sides.^64^ Another advantage of amygdalin therapy appears to be the possibility of pain and inflammation relief.^56^ Chang et al.^68^ established amygdalin's anti-inflammatory and analgesic effects using in vitro cultivation of mouse BV2 microglial cells in 2005. Huang confirmed these effects in vitro on LPS-treated RAW 264.7 cells and in vivo investigations on mice with carrageenan-induced inflammation.^73^ TNF-a and IL-1b transcription are inhibited by 1 mM amygdalin in vitro.^74^ The study's next phase involved injecting carrageenan into a mouse's ankle joint in an in vivo test.^75^ The weight distribution ratio (WDR), ankle circumference, and expression levels of three molecular indicators of pain and inflammation (c-Fos, TNF-a, and IL-1b) were used to analyze the effects of amygdalin.^76^ Carrageenan has been demonstrated to produce edema and lower WDR, making it a good predictor of pain levels.^77^ The administration of amygdalin resulted in a reduction in WDR, which might imply that amygdalin possesses analgesic characteristics.^78^ A variety of inflammatory mediators can produce swelling in the ankles of tested animals.^79^ The expression of c-Fos, TNF-a, and IL-1b in the spinal cord was considerably reduced by the amygdalin administration in the muscle at 0.005 mg/kg.^80^ The reduction of Fos (carrageenan-induced) expression in the spinal cord by amygdalin makes it an attractive option for treating inflammation and pain alleviation in this study.^81^ Treatment with amygdalin has been found to reduce LPS-induced cell inflammation in vitro as well as carrageenan-induced inflammation and edema in mice.^82^ The c-Fos, TNF-a, and IL-1b expression in the spinal cord is considerably reduced by this therapy.^19^ As a result, amygdalin's action is linked to a reduction in proinflammatory cytokine output.^83^ In lipopolysaccharide (LPS)-induced cell line and a mouse model of carrageenan-induced arthritis, amygdalin's analgesic and anti-inflammatory properties were investigated.^84^ Amygdalin, a compound isolated from rosemary fruit seeds, lowers hyperalgesia by inhibiting the production of major pain and inflammatory molecular markers such as necrosis factor (TNF-) and interleukin-1 (IL-1).^85^ Amygdalin isolated from Prunus Armeniacae has also been demonstrated to reduce formalin-induced pain in rats at dosages less than 1 mg/kg, which may be due to its influence on the production of the inflammatory cytokines such as interleukin-1 beta (IL-1β), and tumor necrosis factor α (TNFα-), two main types of cytokines produced by immune cells.^86^ Furthermore, it inhibits the production of cyclooxygenase (COX-2) and nitric oxide synthase (iNOS), lowering E2 prostaglandins and nitric oxide levels, resulting in an anti-inflammatory and analgesic action.^87^

## 8 Amygdalin's effect on the digestive system

The amygdalin role in the intestinal wall and the accompanying risk of food intake has been demonstrated by degrading amygdalin in human gastrointestinal fluids and its metabolite absorption in the small intestine in the GIT gastrointestinal tract and culture of the human intestinal cells.^88^ Amygdalin-derived benzaldehyde can interfere with gastrointestinal function by inhibiting pepsin action.^89^ On the contrary, pepsin suppresses ALT and enhances globalization time in CCl4-treated rats when given at a dosage of 500 mg/kg AST.^90^ Moreover, in the presence of pepsin hydrolysis of almond juice, rat liver connective tissue proliferates less, even though this is unaffected by proliferation owing to the recovery of AST and ALT^91^ levels following di-galactosamine induction.^30^ Amygdalin has also been shown to effectively treat chronic gastritis in rats with atrophic gastritis.^92^ By mimicking a laboratory model of the human gastrointestinal system, Shim et al.^51^ could simulate amygdalin digestion. Digestive enzymes degraded oral-administered amygdalin to prunasin and glucose.^93^ Then, prunasin was broken down into mandelonitrile in the human small intestine, containing beta-glucosidase, to generate hydroxy-mandelonitrile without benzaldehyde, according to the researchers.^31^ This finding implies that gut bacteria may influence amygdalin toxicity.^47^

## 9 Effect of amygdalin on neurodegenerative diseases

According to Cheng et al.,^49^ amygdalin can be effective in treating neurodegenerative disorders like Parkinson's disease. In addition to shielding cells against 6-hydroxydopamine-induced neurotoxicity, it enhances neuronal development by Calreticulin expression stimulation.^48^

## 10 Effect of amygdalin on the genitourinary system

Amygdalin is well-known for its potent anti-fibrotic properties, and it can be utilized to treat renal fibrosis patients. When cultured interstitial fibroblasts are given amygdalin, their capacity to proliferate is reduced, and their generation of growth factor (TGF-β1) is altered.^49^ In addition, when amygdalin was given to animals with obstructive neuropathy, the formation of the extracellular matrix following urinary tract blockage was rapidly reduced. On day 21, amygdalin also appeared to minimize renal damage.^48^ As a result, amygdalin can weaken renal fibroblasts and cause interstitial renal fibrosis in rats.^30^ Estradiol-17 beta is released from ovarian granulosa cells when exposed to various dosages of amygdalin, but progesterone secretion was not observed.^41^ In pig ovaries, amygdalin also controls steroid production.^47^ In addition, the medicinal components of the raw material Keishi-bukuryo-gan, a Japanese herbal medication used to stimulate ovulation in infertile women, include amygdalin. In both in vivo and in vitro circumstances, Keishi-bukuryo-gan promoted steroidogenesis in pre-ovulatory follicles and corpus luteum in rat ovaries.^46^ Hence, the natural component found in bitter almond kernels may decrease FSH and may play a role in folliculogenesis in rabbit ovaries.^45^ However, intramuscular and oral administration of amygdalin significantly affects some endocrine regulators' plasma levels (progesterone, estradiol-17 beta, testosterone), thyroid (triiodothyronine, thyroxine, stimulating thyroid hormone), and hormones (triiodothyronine, thyroxine, stimulating thyroid hormone).^44^ The average body weights and the anterior pituitary glands (prolactin, luteinizing hormone) of rabbits used in the investigation were unaffected.^43^

## 11 Amygdalin's anticancer activity

Amygdalin is thought to be found in several plants’ seeds, namely apricots, almonds, apples, and peaches, allowing it to be tested on a wide range of cancer cells.^32^ Chen et al.^49^ discovered that amygdalin had an apoptotic impact on cervical cancer Hela cells for the first time. The amygdalin-treated peach cell line was stained with 4, 6-diamino-2-phenyl indole (DAPI) before being treated with annexin V-FITC and propidium iodide.^42^ So, the BCL-2 anti-apoptotic protein function diminishes, but the Bax protein function rises.^41^ There was also an increase in caspase activity and the initiation of innate apoptotic pathways.^40^ The lifespan of HeLa cells in vitro was decreased due to using amygdalin, suggesting that it may have a therapeutic impact on cervical cancer cells. An identical finding has also been observed in vivo.^39^

Furthermore, the impact of amygdalin isolated from Armenlacae semen, a prunasin family member, has been studied on prostate cancer cells DU 145 and LN CAT.^37^ With the decreased expression of anti-apoptotic BCL-2 protein and increased Bax protein expression, an increase in caspase-3 enzyme has been found.^94^ As anti-apoptotic BCL-2 protein expression was decreased and Bax protein expression was increased, an increase in the caspase-3 enzyme was found.^84^ Amygdalin extract is thought to trigger prostate cancer cells death in humans by apoptosis.^85^

Amygdalin's chemical inhibitory capability has also been studied in vitro in breast cancer cells. It can also produce cytotoxicity in MCF7 (ER) cells, MDA MB-231 cells, and Hs 578T cells (triple-negative breast cancer cell lines (TNBC)). BCL-2, Bax, and caspases have all been shown to have similar effects.^86^ The adherence of Hs 578T TNBC cells was also reduced by mitogen-activated protein kinase (p38 MAPK) and apoptotic signaling molecule with additional treatment with amygdalin.^87^ Amygdalin indicated anticancer properties regarding breast cancer cells.^30^ A similar example of an adhesion pattern was seen in bladder cancer cells. Besides, UMUC-3 tumor cells, RT112 cells, and TCCSUP cells all showed a significant reduction in adhesion after 24 h or two weeks of amygdalin administration.^79^ The first two cancer cell lines exhibited decreased migration, whereas TCCSUP exhibited enhanced migration. It was claimed that amygdalin's anticancer action is limited to certain types of cancer cells.^78^ A similar thing happened with cervical cancer cells, where amygdalin had a therapeutic impact on the HeLa cell line but not on FL cells. It has been found that converting amygdalin to neoamygdalin in an aqueous solution inhibits its anticancer effect in promyelocytic leukemia cells. The amygdalin extract from Persicae semen was shown to be the active form of D by HPLC chromatography. Thus, the extract was boiled to avoid epimerization before being used for promyelocytic leukemia cells (HL-60). Increased cell death has also been recorded, suggesting that apoptosis is taking place.^77^

**Fig. 3:**
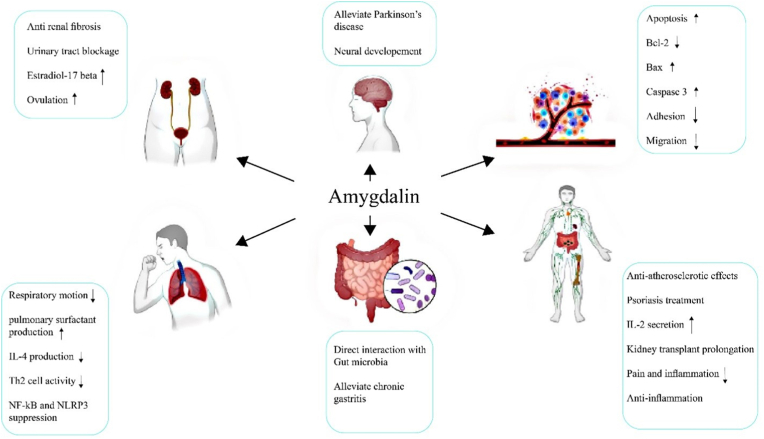
potential pharmaceutical consumption of amygdalin in the clinics.

Moreover, physical alterations in the nucleus, as well as cell DNA fragmentation, have been detected. In addition, amygdalin's therapeutic activity on H1299 and PALM cells with non-small cell lung cancer (NSCLC) has been observed in vitro.^76^ Inhibition of cell proliferation occurred at high concentrations of amygdalin, and inhibition of cell migration and invasion occurred at low concentrations. Amygdalin has been shown to reduce the number of mature microtubules in the aortic ring of amygdalin-treated diabetic rats, inhibiting streptozotocin-induced endothelial cells in streptozotocin-induced diabetic rat endothelial cells.^75^ After seven days of incubation, the rat aortic ring moved and multiplied without amygdalin therapy. It has also been proposed that amygdalin's anti-angiogenic properties may contribute to its tumor-suppressive properties and the potential therapeutical roles of amygdalin are shown in Fig. 3.^92^

## 12 Effect of intestinal microbial flora on amygdalin

GIT, lumen, intestinal, and intestinal microorganisms impact the metabolism of medicines and other foreign chemicals that enter the body, especially when taken orally. Firmicutes, bacteroids, and actinobacteria are the most common anaerobic bacteria that help to release cyanide in the gut.^30^ Nucleases, lipases, transferases, peptidases, and microflora enzymes are among the numerous enzymes found in the intestine. It has been discovered that intestinal beta-glucosidase and microbial beta-glucosidase create distinct products.^79^ Beta-glucosidase, also known as lactase fluorescein hydrolase (LPH) and cytosolic beta-glucosidase (CBG), is a glycosidic bond destroyer that operates on other substances, including bile. No hydrocyanic acid is generated when amygdalin is converted to prunasin by intestinal beta-glucosidase. Another gut microbe, beta-glucosidase, converts amygdalin to HCN.^75^ Intestinal bacteria have been demonstrated in several investigations, in which amygdalin is hydrolyzed to cyanide, which is proportional to the microbial composition in the gut.^74^ Besides, when intestinal microbial growth is promoted in mice supplied at 300 mg/kg, amygdalin hydrolysis toxicity is reduced, but it produces toxicity and greater mortality in untreated mice.^68^

Comparing mice and monkeys, human feces have been demonstrated to hydrolyze roughly 50% of amygdalin to cyanide due to the presence of abundant microbial flora. Bacteroids are mostly used to produce glucosidase. The bacterial population in the gut is affected and regulated by prebiotics and probiotics.^64^^,^^73^^,^^74^ Besides, prebiotics are biological molecules, whereas probiotics are organisms that control the actions of the gut microbiota for the host's benefit. Prebiotics can bind or absorb carcinogens, lowering the danger of amygdalin-induced cyanide poisoning. Bactericidal beta-glucosidase activity was reduced by Lactobacillus and Bifidobacterium.^56^^,^^85^^,^^90^

## 13 Amygdalin effect on cell cycle progression and apoptosis

Amygdalin also has anticancer properties because it affects or modifies proteins involved in the cell cycle. Aside from triggering apoptosis in PC3 and LNCaP cells in prostate cancer after 24 h and two weeks of therapy, amygdalin has also been shown to increase G0/G1 phase cells and reduce G2/M phase cells. Modulation of some cell cycle proteins, such as cyclin-dependent kinases (Cdks), has also been reported.^35^^,^^70^^,^^71^ In amygdalin-treated SNu-C4 cells, colorectal carcinoma cell line, the identical scenario was observed, in which certain cycle proteins, primarily exonuclease, topoisomerase, and binding protein, were regulated. The researchers reduced protein expression using cDNA microarray analysis, and they also observed a decrease in mRNAs expression level using RT-PCR analysis.^64^^,^^65^^,^^72^ Amygdalin has been proven to lessen the expression of cell cycle-related genes in SNU-C4 colorectal cancer cells, such as exonuclease 1 (EXO1), topoisomerase (DNA) I (TOP1), subfamily F, and ATP binding cassette. Therefore, it can impact the tumor cell cycle, prevent cell proliferation and show its anti-tumor effect. It can be concluded that amygdalin can control the cell cycle and limit cell proliferation by modulating cell cycle proteins or genes, and in particular, amygdalin can reduce malignant tumor cell proliferation, particularly in prostate and colon cancer.^55^^,^^64^, ^65^, ^66^

**Fig. 4:**
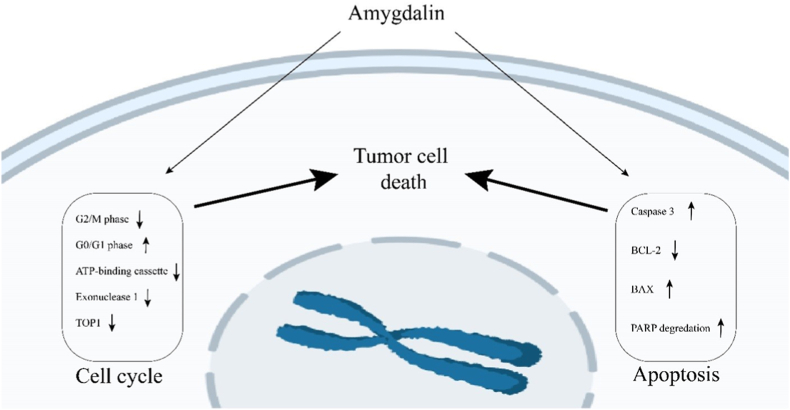
The amygdalin impact on cell cycle and apoptosis and ultimately on tumor cell death.

Chen et al.^49^ discovered that amygdalin might cause apoptosis in DAPI-impregnated HeLa cells by boosting caspase-3 expression, which lowered the expression of BCL-2 and elevated the expression of Bax in HeLa cells treated by amygdalin, indicating an essential path involved in initiating amygdalin-based apoptosis. It can also cause apoptosis in prostate cancer cells.^14^^,^^16^^,^^17^ After treatment with amygdalin, Chang et al. discovered that Bax enzyme expression and caspase-3 activity were elevated, whereas BCL-2 expression dropped in DU145 and LNCaP cells, resulting in apoptosis in prostate cancer cells.^8^ Lee et al.^15^ evaluated apoptotic proteins expression in amygdalin-treated breast cancer cells with various dosages. They discovered that amygdalin raised the expression of the proapoptotic Bax protein while decreasing the expression of the anti-apoptotic protein BCL-2 Procaspase 3. PARP degradation was also seen in amygdalin-treated breast cancer cells at the same time.^8^ These findings imply that amygdalin inhibits tumor growth by inducing apoptosis and modulating apoptosis-related proteins, which is significant in prostate and cervical cancer cells (Fig. 4).^74^, ^75^, ^76^, ^77^

## 14 Association between amygdalin and Β-glucosidase

Beta-glucosidases (-D-glucopyrranoside glucohydrolase) [E.C.3.2.1.21] are enzymes that hydrolyze carbohydrates' glycosidic bonds, releasing end-glycosyl residues, glycosides, and oligosaccharides. They exist in a wide range of species, including eukaryotes, archaea, and bacteria.^30^ They are also engaged in key activities such as biomass conversion, glycolipid breakdown, lignification, pest defense, phytohormone activation, and cell wall catabolism. Gaucher disease (induced by beta-glucosidase deficiency), in which glycosides accumulate in lysosomal tissues, is likewise treated with beta-glucosidases. Beta-glucosidases are mostly employed as a cell plant for cellulose hydrolysis on a commercial scale.^90^ At temperatures up to 90 °C, heat-resistant glucosidases are utilized to manufacture glucose from cellulose, and isoflavones can also make beneficial aglycones. Beta-glucosidase, also known as emulsion, is present in edible plants such as apricot kernels (which are also high in antioxidants), almond kernels, mushrooms, lettuce, and green pepper, and is produced by microorganisms such as black aspergillus, presented in the gut microbiota.

Plants, fungi, and bacteria all have beta-glucosidase identical in sequence and structure. The activity of the substrate or the identity of their nucleotide sequences can be used to classify them.^88^ Beta-glucosidases can be categorized into three classes according to the substrate nature: (1) aryl beta-glucosidases (which have a significant preference for aryl-beta-glucosides), (2) cellulose (which hydrolyzes only disaccharides), and (3) glucosidases with Extensive Specificity (shows activity on many types of substrates and are common beta-glucosidases). Beta-glucosidases are subdivided into two subfamilies based on sequence homogeneity: BGA, which includes beta-glucosidases and phospho-beta-glucosidase (from bacteria to mammals), and BGB, which only contains beta-glucosidases (from yeasts, molds, and bacteria).^86^ An alternate categorization framework according to the amino acid sequence and structural similarity was also established for glycoside hydrolase. Enzymes in a single family with broad amino acid sequence similarity and completely protected sequences are evaluated in this method.^87^ The Carbohydrate Active enzyme (CAZY) website (http://www.cazy.org) now lists 133 glycoside hydrolase (GH) families.^84^ The majority of these families are divided into subgroups. One grouping consists of families with protected catalytic amino acids, identical catalytic domain structures, and a shared catalytic race and mechanism. The GH-A family has the most members, including beta-glucosidases from the GH1, GH5, and GH30 families.^81^ The GH1 family has the most known beta-glucosidases. Glucosidases from bacteria, plants, and animals belong to the GH1 family, whereas glucosidases from bacteria, mold, and yeast belonging to the GH3 family.^73^ The GH family is divided into structural families by the International Union of Biochemistry and Molecular Biology. As it is difficult to define the whole spectrum of substrates for specific enzymes, this categorization scheme, based on enzymes' structural features, is more valuable than the substrate property.^74^ Using bioinformatics tools and the biological method of the third structure system, structural traits of one family help analyze the structure of other members of the same family, particularly in the active position, enzyme mechanism, and substrate characteristics. The evolution of glycoside hydrolase is also explained by the categorization of enzyme families.^73^

When beta-glucosidase activates amygdalin, hydrocyanic acid is produced, which limits cellular respiration and causes cell death (Fig. 2). Cancer cells have a lower sulfur hydroxylase concentration than healthy ones. As a result, these cells’ ability to detoxify the amygdalin hydrolysis-released hydrocyanic acid is restricted. The anticancer action of amygdalin is enhanced when it is combined with beta-glucosidase.^56^ In cancer cells, anaerobic glycolysis is the most common route. Acidic circumstances boost beta-glucosidase activity, which causes more hydrocyanic acid and benzaldehyde to be produced in cancer cells, resulting in a fatal impact. Hydrocyanic acid is a non-specific agent compared to other low molecular weight medications since it diffuses due to the toxicity of cyanide and causes its removal.^76^ According to Makarević et al.,^29^ bladder cancer cells were unaffected by amygdalin concentrations of less than 15 mmol/L. Nevertheless, stimulation of beta-glucosidase reduced cell growth and migration. Apoptosis increased in a dose-dependent manner, and cells terminated in the S phase.^77^

## 15 Amygdalin and beta-glucosidase in the treatment of cancer

Amygdalin has been employed as a cancer therapy adjuvant because of its anti-tumor properties. Amygdalin has been demonstrated to help people with advanced cancer symptoms and extend their lives. Amygdalin can manage and alleviate pleural or lateral carcinoma to some extent.^76^ In vitro, amygdalin hydrolytic products, hydrocyanic acid, and benzaldehyde show anticancer effects. Hydrocyanic acid destroys tumor cells by preventing the formation of adenosine triphosphate and inhibiting CCO, the terminal enzyme in the mitochondrial electron transport chain, due to the activity of beta-glucosidase on amygdalin.^77^ The use of a combination of specific beta-glucosidase activators with amygdalin has been evaluated to treat mice with implanted tumors.^76^ Amygdalin can also be used in combination with other medications to treat skin cancer. Treatment of LoVo cells with a combination of amygdalin (0.1, 0.5, and 1.1 mmol/L) and beta-glucosidase (250 nmol/L) for 24 h promoted apoptosis and necrosis, according to one research. The characteristic morphological alterations that occur during apoptosis have been confirmed using DNA gel electrophoresis and flow cytometry.^75^ As a result, amygdalin might be a novel factor in tumor treatment by promoting apoptosis. Amygdalin has also been used to treat colon cancer as a prodrug. Amygdalin is considered a natural prodrug-producing cyanide that promotes Bax expression and caspase-3 activity in LoVo cells, both of which are implicated in apoptosis.^90^

The primary idea of enzyme therapy with prodrug therapy in treating cancers using a particular antibody-beta-glucosidase complex using a cross-linking agent and an activating enzyme in vitro is to use a particular antibody-beta-glucosidase complex with an activating enzyme and a cross-linking agent.^89^ Cancer cell surface antigens will be detected by beta-glucosidase using antibodies once this combination is given intravenously. The prodrug Amygdalin is activated by beta-glucosidase and attaches to the tumor's target location, giving it the ability to destroy it. Lian and colleagues developed a mouse model of human colorectal cancer. An anti-CEA McAb beta-glucosidase molecule was injected into the rats' tail veins, followed by 50 mg/kg amygdalin three times a week for six weeks.^19^^,^^84^, ^85^, ^86^^,^^88^ Before and after injection, tumor inhibition, and tumor volume were determined. Hence, pathological alterations in mice tumor tissues and primary organs were identified.^90^ Their findings in a rat model of colorectal cancer imply that a conjugated amygdalin prodrug system combined with the anti-CEA McAb beta-glucosidase molecule significantly limits xenograft development.^84^ The primary organs, on the other hand, showed no signs of cytotoxicity. Cell membrane peptides are polypeptides^1^ with 30 amino acids or less that may transport a wide range of biological compounds into the cell.^88^ As a result, these cell membrane peptides compensate for the shortcomings of enzyme-prodrug treatment.^90^ The binding of cell membrane peptides to beta-glucosidase promotes the enzyme's penetration into capillary endothelial cells and deep extracellular solid tumors, resulting in tumor cell death.^87^

## 16 Conclusion

Amygdalin has been suggested as a therapy for a wide variety of medical issues, including leukoderma, leprosy, bronchitis, nausea, and cough. Laboratory and animal studies have confirmed the benefits for digestion and reproduction, as well as reductions in neurotoxicity, cardiac hypertrophy, and glucose levels. Previous research has mainly focused on amygdalin's pharmacological efficacy and safety, so our knowledge of the molecular pathways underlying its actions is still in its infancy. As there is currently a lack of data to back up this treatment, it should be pursued with caution. Little is known about amygdalin's pharmacokinetics or its systemic toxicity. Innovative studies are required to determine the compound's therapeutic potential, safety, and adverse effects. More study is required to fully understand the health effects. Some examples include overactive hearts, diabetes, inflammation, digestive issues, neurodegeneration, and reproduction. Although there is now more information accessible than ever before, it is not yet adequate for conclusive analysis. Recent research has shown that taking amygdalin orally is more dangerous than injecting it. Despite promising in vitro results from amygdalin-nanocarrier coupling, further in vivo confirmation of these findings is required. Clinical trials have shown that amygdalin/-Glu MDEPT is an effective strategy. Amygdalin's therapeutic benefits and side effects could be enhanced with further research into its encapsulation and anti-cancer potential.

## Data availability

The data used to support the findings of this study are available from the corresponding author upon reasonable request.

## Declaration of competing interest

The authors declare no conflicts of interest.

## References

[1] Makarević J., Tsaur I., Juengel E. (2016). Amygdalin blocks bladder cancer cell growth in vitro by diminishing cyclin A and cdk2. PLoS One.

[2] Boehm S.M.E.L., Milazzo S., Horneber M., Ernst E., Stefania MilazzoMarkus HorneberEdzard Ernst (2017). Laetrile treatment for cancer. Br Biomed Bull.

[3] Jamshidzadeh A., Rasekh H.R.R., Amin L.M. (2001). Rhodanese and arginase activity in normal and cancerous tissues of human breast, esophagus, stomach and lung. Arch Iran Med.

[4] Moon J.Y.Y., Kim S.W.W., Yun G.M.M. (2015). Inhibition of cell growth and down-regulation of telomerase activity by amygdalin in human cancer cell lines. Anim Cell Syst.

[5] Chen C., Xu F., Yuan S. (2022). Competing risk analysis of cardiovascular death in patients with primary gallbladder cancer. Cancer Med.

[6] Syrigos O.N., Rowlinson-Busza G., Epenetos A.A., Syrigos K.N., Rowlinson‐Busza G., Epenetos A.A. (1998). In vitro cytotoxicity following specific activation of amygdalin by β‐glucosidase conjugated to a bladder cancer‐associated monoclonal antibody. Int J Cancer.

[7] Chang H.K., Shin M.S., Yang H.Y. (2006). Amygdalin induces apoptosis through regulation of Bax and bcl-2 expressions in human DU145 and LNCaP prostate cancer cells. Biol Pharm Bull.

[8] Newton G.W., Schmidt E.S., Lewis J.P., Lawrence R., Conn E. (1981). Amygdalin toxicity studies in rats predict chronic cyanide poisoning in humans. West J Med.

[9] Torre L.A., Siegel R.L., Ward E.M., Jemal A. (2016). Global cancer incidence and mortality rates and trends—an update. Cancer Epidemiol Prev Biomarkers.

[10] Rayan A., Raiyn J., Falah M. (2017). Nature is the best source of anticancer drugs: indexing natural products for their anticancer bioactivity. PLoS One.

[11] Makarević J., Tsaur I., Juengel E. (2016). Amygdalin delays cell cycle progression and blocks growth of prostate cancer cells in vitro. Life Sci.

[12] Ogata K., Volini M. (1990). Mitochondrial rhodanese: membrane-bound and complexed activity. J Biol Chem.

[13] Kožich V., Ditrói T., Sokolová J. (2019). Metabolism of sulfur compounds in homocystinurias. Br J Pharmacol.

[14] Cipollone R., Ascenzi P., Tomao P., Imperi F., Visca P. (2008). Enzymatic detoxification of cyanide: clues from Pseudomonas aeruginosa Rhodanese. J Mol Microbiol Biotechnol.

[15] Ayaz Z., Zainab B., Khan S. (2020). In silico authentication of amygdalin as a potent anticancer compound in the bitter kernels of family Rosaceae. Saudi J Biol Sci.

[16] Kleessen B., Sykura B., Zunft H.J., Blaut M. (1997). Effects of inulin and lactose on fecal microflora, microbial activity, and bowel habit in elderly constipated persons. Am J Clin Nutr.

[17] Albogami S., Hassan A., Ahmed N. (2020). Evaluation of the effective dose of amygdalin for the improvement of antioxidant gene expression and suppression of oxidative damage in mice. PeerJ.

[18] Mani J., Rutz J., Maxeiner S. (2019). Cyanide and lactate levels in patients during chronic oral amygdalin intake followed by intravenous amygdalin administration. Compl Ther Med.

[19] long Li Y., xing Li Q., jiang Liu R., qian Shen X. (2018). Chinese medicine Amygdalin and β-glucosidase combined with antibody enzymatic prodrug system as a feasible antitumor therapy. Chin J Integr Med.

[20] Aminlari M., Malekhusseini A., Akrami F., Ebrahimnejad H. (2007). Cyanide-metabolizing enzyme rhodanese in human tissues: comparison with domestic animals. Comp Clin Pathol.

[21] Shim S.M., Kwon H. (2010). Metabolites of amygdalin under simulated human digestive fluids. Int J Food Sci Nutr.

[22] Zagrobelny M., Bak S., Rasmussen A.V., Jørgensen B., Naumann C.M., Møller B.L. (2004). Cyanogenic glucosides and plant-insect interactions. Phytochemistry.

[23] Kalaiyarasan G., Veerapandian M., Jebamercy G., Balamurugan K., Joseph J. (2019). Amygdalin-functionalized carbon quantum dots for probing β-glucosidase activity for cancer diagnosis and therapeutics. ACS Biomater Sci Eng.

[24] Al-Khafaji K., Taskin Tok T. (2021). Understanding the mechanism of amygdalin's multifunctional anti-cancer action using computational approach. J Biomol Struct Dyn.

[25] Hwang H.J., Kim P., Kim C.J. (2008). Antinociceptive effect of amygdalin isolated from Prunus armeniaca on formalin-induced pain in rats. Biol Pharm Bull.

[26] Chen Y.U., Ma J., Wang F. (2013). Amygdalin induces apoptosis in human cervical cancer cell line HeLa cells. Immunopharmacol Immunotoxicol.

[27] Luo H., Zhao F., Zhang F., Liu N. (2018). Influence of amygdalin on PDG, IGF and PDGFR expression in HSC-T6 cells. Exp Ther Med.

[28] Blaheta R.A., Nelson K., Haferkamp A., Juengel E. (2016). Amygdalin, quackery or cure?. Phytomedicine.

[29] Makarević J., Tsaur I., Juengel E. (2014). Amygdalin blocks bladder cancer cell growth in vitro by diminishing cyclin A and cdk2. PLoS One.

[30] Abboud M.M., Al Awaida W., Alkhateeb H.H., Abu-Ayyad A.N. (2019). Antitumor action of amygdalin on human breast cancer cells by selective sensitization to oxidative stress. Nutr Cancer.

[31] Zhou J., Hou J., Rao J., Zhou C., Liu Y., Gao W. (2020). Magnetically directed enzyme/prodrug prostate cancer therapy based on β-glucosidase/amygdalin. Int J Nanomed.

[32] Qadir M., Fatima K. (2017). Review on pharmacological activity of amygdalin. Arch Cancer Res.

[33] Makarević J., Rutz J., Juengel E. (2014). Amygdalin blocks bladder cancer cell growth in vitro by diminishing cyclin A and cdk2. PLoS One.

[34] Dang T., Nguyen C., Tran P.N. (2017). Physician beware: severe cyanide toxicity from amygdalin tablets ingestion. Case Rep Emerg Med.

[35] Liczbiński P., Bukowska B. (2018). Molecular mechanism of amygdalin action in vitro: review of the latest research. Immunopharmacol Immunotoxicol.

[36] Moertel C.G., Fleming T.R., Rubin J. (1982). A clinical trial of amygdalin (Laetrile) in the treatment of human cancer. N Engl J Med.

[37] Qadir M., Fatima K. (2017). Review on pharmacological activity of amygdalin. Arch Cancer Res.

[38] Cao H., Sethumadhavan K., Cao F., Wang T.T.Y. (2021). Gossypol decreased cell viability and down-regulated the expression of a number of genes in human colon cancer cells. Sci Rep.

[39] Yulvianti M., Zidorn C. (2021). Chemical diversity of plant cyanogenic glycosides: an overview of reported natural products. Molecules.

[40] Mosayyebi B., Imani M., Mohammadi L. (2021). Comparison between β-cyclodextrin-amygdalin nanoparticle and amygdalin effects on migration and apoptosis of MCF-7 breast cancer cell line. J Cluster Sci.

[41] Kolesarova A., Baldovska S., Roychoudhury S. (2021). The multiple actions of amygdalin on cellular processes with an emphasis on female reproduction. Pharm Times.

[42] Jaszczak-Wilke E., Ż Polkowska, Koprowski M., Owsianik K., Mitchell A.E., Amygdalin Bałczewski P. (2021). Toxicity, anticancer activity and analytical procedures for its determination in plant seeds. Mol.

[43] Cao H., Sethumadhavan K., Cao F., Wang T.T.Y. (2021). Gossypol decreased cell viability and down-regulated the expression of a number of genes in human colon cancer cells. Sci Rep.

[44] Yulvianti M., Zidorn C. (2021). Chemical diversity of plant cyanogenic glycosides: an overview of reported natural products. Molecules.

[45] Wang R., Zhang D., Tang D. (2021). Amygdalin inhibits TGFβ1-induced activation of hepatic stellate cells (HSCs) in vitro and CCl4-induced hepatic fibrosis in rats in vivo. Int Immunopharm.

[46] Kenar J.A., Cao H., Sethumadhavan K. (2021). Advances in colon cancer research: in vitro and animal models. Curr Opin Genet Dev.

[47] Omelka R., Kovacova V., Mondockova V., Grosskopf B., Kolesarova A., Martiniakova M. (2021). Cyanogenic glycoside amygdalin influences functions of human osteoblasts in vitro. J Environ Sci Heal Part B.

[48] Abboud M.M., Al Awaida W., Alkhateeb H.H., Abu-Ayyad A.N. (2019). Antitumor action of amygdalin on human breast cancer cells by selective sensitization to oxidative stress. Nutr Cancer.

[49] Lannagan T.R.M., Jackstadt R., Leedham S.J., Sansom O.J. (2021). Advances in colon cancer research: in vitro and animal models. Curr Opin Genet Dev.

[50] Yang J., Ren Z., Du X., Hao M., Zhou W. (2014). The role of mesenchymal stem/progenitor cells in sarcoma: update and dispute. Stem cell Investig.

[51] Afzaljavan F., Tavakol Afshari J., Ahmad Mohammed Alwan (2021). The impact of CYP19A1 variants and haplotypes on breast cancer risk, clinicopathological features and prognosis. Mol Genet Genomic Med.

[52] Aghadavod E., Shi J., Chen Q. (2019). Recent updates and future perspectives about amygdalin as a potential anticancer agent: a review. Cancer Med.

[53] Widders A., Broom A., Broom J. (2020). SARS-CoV-2: the viral shedding vs infectivity dilemma. Infect Dis Heal.

[54] Long Q.X., Tang X.J., Shi Q.L. (2020). Clinical and immunological assessment of asymptomatic SARS-CoV-2 infections. Nat Med.

[55] Zhang C., Zhang D., Wang Y. (2022). Pharmacokinetics and anti-liver fibrosis characteristics of amygdalin: key role of the deglycosylated metabolite prunasin. *Egypt J Histol*. Published online.

[56] Karsten K. (2022).

[57] Cevik M., Tate M., Lloyd O., Maraolo A.E., Schafers J., Ho A. (2020). SARS-CoV-2, SARS-CoV, and MERS-CoV viral load dynamics, duration of viral shedding, and infectiousness: a systematic review and meta-analysis. The Lancet Microbe.

[58] Salama R., Ramadan A., Alsanory T., Herdan M., Fathallah O., Alsanory A. (2019). Experimental and therapeutic trials of amygdalin. Int J Biochem Pharmacol.

[59] Elbastawisy Y.M., Mohamed H.A. (2022). Therapeutic effect of amygdalin on acetic acid-induced colitis in rats: histopathological and immunohistochemical study. Egypt Acad J Biol Sci D Histol Histochem.

[60] Syriac A.K., Nandu N.S., Leone J.P. (2022). Central nervous system metastases from triple-negative breast cancer: current treatments and future prospective. Breast Cancer.

[61] Wang R., Zhang D., Sun K. (2021). Amygdalin promotes the activity of T cells to suppress the progression of HBV-related hepatocellular carcinoma via the JAK2/STAT3 signaling pathway. BMC Infect Dis.

[62] Jiagang D., Li C., Wang H. (2011). Amygdalin mediates relieved atherosclerosis in apolipoprotein E deficient mice through the induction of regulatory T cells. Biochem Biophys Res Commun.

[63] El-Kholy W.B., Abdel-Rahman S.A., El F.N.A.E.H., Issa N.M. (2021). Effect of vitamin B17 on experimentally induced colon cancer in adult male albino rat. Folia Morphol (Wars.).

[64] Motawea S.M., Youssef S.A.A., Abdel-Aleem G.A., Mohamed M.F., Mostafa M.S. (2022). Effect of amygdalin (vitamin B17) on induced mammary tumor in virgin female albino rats: histological and morphometric study. Egypt J Histol.

[65] Ozkan S. Psychosocial aspects of breast cancer. Glob Perspect Cancer Care Relig Spirituality, Cult Divers Heal Heal. Published online 2022:302.

[66] Song Z., Xu X. (2014). Advanced research on anti-tumor effects of amygdalin. J Cancer Res Therapeut.

[67] Ryser M.D., Lange J., Inoue L.Y.T. (2022). Estimation of breast cancer overdiagnosis in a US breast screening cohort. *Ann Intern Med*. Published online.

[68] Zhang C., Zhang D., Wang Y. (2022). Pharmacokinetics and anti-liver fibrosis characteristics of amygdalin: key role of the deglycosylated metabolite prunasin. *Phytomedicine*. Published online.

[69] Wang R., Zhang D., Tang D. (2021). Amygdalin inhibits TGFβ1-induced activation of hepatic stellate cells (HSCs) in vitro and CCl4-induced hepatic fibrosis in rats in vivo. Int Immunopharm.

[70] Anjum N., Sheikh M.A., Saini C.S., Hameed F., Sharma H.K., Bhat A. (2022). Handbook of Plant and Animal Toxins in Food.

[71] Shastri A.A., Lombardo J., Okere S.C. (2022). Personalized nutrition as a key contributor to improving radiation response in breast cancer. Int J Mol Sci.

[72] Luo Y., Zhou T. (2022). Connecting the dots: targeting the microbiome in drug toxicity. Med Res Rev.

[73] Haghdoust M., Serajkhorrami N., Makvandi B. (2022). The effectiveness of mindfulness based on stress managment on death anxiety, disaster of imagination, acceptance and severity of pain in prostat cancer patients. J Ilam Univ Med Sci.

[74] Jaswal V., Palanivelu J., Ramalingam C. (2018). Effects of the Gut microbiota on amygdalin and its use as an anti-cancer therapy: substantial review on the key components involved in altering dose efficacy and toxicity. Biochem Biophys reports.

[75] Hönigova K., Navratil J., Peltanova B., Polanska H.H., Raudenska M., Masarik M. (2022). Metabolic tricks of cancer cells. Biochim Biophys Acta Rev Cancer.

[76] Zhang T., Yang S., Zhang B., Yang D., Lu Y., Du G. (2022).

[77] He X.Y., Wu L.J., Wang W.X., Xie P.J., Chen Y.H., Wang F. (2020). Amygdalin - a pharmacological and toxicological review. J Ethnopharmacol.

[78] Naveena K., Chinniah C., Shanthi M. (2021). Cyanogenic glycosides and plant-herbivore interactions. J Entomol Zool Stud.

[79] Mizokami A., Nishimoto K., Matsuyama H. (2022). Efficacy of new therapies for relapse after docetaxel treatment of bone metastatic castration-resistant prostate cancer in clinical practice. Anticancer Res.

[80] Ellinger J., Alajati A., Kubatka P. (2022). Prostate cancer treatment costs increase more rapidly than for any other cancer—how to reverse the trend?. *EPMA J*. Published online.

[81] Nishimoto K., Nakajima K., Oyama M. (2022).

[82] Zhou J., Hou J., Rao J., Zhou C., Liu Y., Gao W. (2020). Magnetically directed enzyme/prodrug prostate cancer therapy based on β-Glucosidase/Amygdalin. Int J Nanomed.

[83] Halenár M., Medveďová M., Maruniaková N., Kolesárová A. (2021). Amygdalin and its effects on animal cells. J Microbiol Biotechnol Food Sci.

[84] Congcong Z., Jiacheng L., Chao Z. (2022). Amygdalin protects against acetaminophen-induced acute liver failure by reducing inflammatory response and inhibiting hepatocyte death. *Biochem Biophys Res Commun*. Published online.

[85] Aydın D., Özkan K., Aydın A. (2021). The combination of amygdalin with some anticancer, antiparasitic, and antigout drugs against MG63, Saos2, SW1353, and FL cells in vitro. J Med Food.

[86] Abudoukelimu A., Yang X., Ge L., Zeng X., Shu Y., Zhao Z. (2022). Effects of amygdalin on TLR4/NF-[KAPPA] B signaling pathway-mediated proliferation and apoptosis of gastric cancer cells. Curr Top Nutraceutical Res.

[87] Liu Z., Liu S., Gao D., Li Y., Tian Y., Bai E. (2022). An optical sensing platform for beta-glucosidase activity using protein-inorganic hybrid nanoflowers. *J Fluoresc*. Published online.

[88] Leapman M.S., Dinan M., Pasha S. (2022). Mediators of racial disparity in the use of prostate magnetic resonance imaging among patients with prostate cancer. JAMA Oncol.

[89] Luo L., Jiang J., Luan H. (2022). Spatial and temporal patterns of prostate cancer burden and their association with Socio‐Demographic Index in Asia, 1990–2019. Prostate.

[90] Deflandre B., Jadot C., Planckaert S. (2022). Structure and function of BcpE2, the most promiscuous GH3-family beta-glucosidase for scavenging glucose from heterosides. bioRxiv.

[91] Alwan A.M., Tavakol A.J. (2022). Investigating the protective role of rhodanese enzyme against cyanide, the cytotoxic by-product of amygdalin, in HDF and L929 cell lines. Lett Drug Des Discov.

[92] Alwan A.M., Afshari J.T., Rokaya D. (2022).

[93] Mohammed Alwan A., Tavakol Afshari J., Afzaljavan F. (2022).

[94] Al-Khafaji K., Taskin Tok T. (2021). Amygdalin as multi-target anticancer drug against targets of cell division cycle: double docking and molecular dynamics simulation. J Biomol Struct Dyn.

